# Prospective association of general anesthesia with risk of cognitive decline in a Chinese elderly community population

**DOI:** 10.1038/s41598-023-39300-5

**Published:** 2023-08-18

**Authors:** Wei Li, Jianjun Jiang, Song Zhang, Ling Yue, Shifu Xiao

**Affiliations:** 1 Department of Geriatric Psychiatry, Shanghai Mental Health Center, Shanghai Jiao Tong University School of Medicine, Shanghai, 200030 China; 2 Alzheimer’s Disease and Related Disorders Center, Shanghai Jiao Tong University, Shanghai, China; 3 Department of Anorectal, KongJiang Hospital of Yangpu District, 480 Shuangyang Road, Shanghai, 200093 China; 4 Department of Anesthesiology, Renji Hospital, Shanghai Jiao Tong University School of Medicine, No. 160 Pujian Road, Shanghai, 200127 China

**Keywords:** Neurological disorders, Human behaviour

## Abstract

As life expectancy increases and the population grows, the number of surgeries performed each year is likely to continue to increase. We evaluated whether surgery with general anesthesia increases risk for cognitive impairment in a Chinese elderly community population. The current data was obtained from the China Longitudinal Aging Study (cohort 1) and Shanghai Brain Aging study (cohort 2). Cohort 1 included 1545 elderly people with normal cognitive function, who underwent a screening process that included physical examination, medical history, baseline and 1-year follow-up assessments of cognitive function by a face-to-face interview. Cohort 2 included an additional 194 elderly people with normal cognitive function, all of whom, unlike cohort 1, underwent T1-phase MR imaging scans. In cohort 1, 127 elderly people with normal cognitive function transformed into mild cognitive impairment, 27 into dementia, while 1391 still maintained normal cognitive function. By using Cox regression analysis, we found that surgery with general anesthesia was a risk factor for cognitive impairment (*p* = 0.013, HR = 1.506, 95% CI 1.091–2.078); In cohort 2, we found that elderly people with a history of surgery with general anesthesia had lower Montreal Cognitive Assessment (MoCA) scores and smaller right amygdala volume (*p* < 0.05). Through correlation analysis, we found that the volume of the right amygdala was significantly correlated (*p* = 0.003, r = 0.212) with MoCA. Then by using the linear regression analysis (mediation model), we found that surgery with general anesthesia directly affected the MoCA score by affecting the volume of the right amygdala (B = 1.315, *p* = 0.036 95% CI 0.088–2.542). We confirm surgery with general anesthesia as a risk factor for cognitive impairment, and its mechanism may be related to its effect on the volume of the right amygdala.

## Introduction

General anesthesia is a state of unconsciousness in which the body feels no pain and is achieved by the use of anesthetic drugs. It has been widely used in certain medical and surgical procedures^1^. There has long been a mistaken belief that general anesthesia is completely reversible and that the central nervous system reverts to its original state once the anesthetic is removed from the active site. However, studies have shown that interference with the normal functioning of these targets can lead to long-term desirable or undesirable effects^2^. For example, there is strong evidence from animal studies that standard doses of conventional anesthetics may produce long-term learning and memory impairments after exposure to anesthesia^3,4^, while in human studies, general anesthesia has also been found to increase the risk of cognitive impairment^5,6^. The possible mechanisms include inflammation, mitochondrial dysfunction, calcium dysregulation, and alterations to tau protein^7^.

In addition to the mechanisms described above, general anesthesia may also lead to cognitive decline by affecting the structure and function of specific brain regions^6,8^. The amygdala is the center of emotional processing, including the processing of anxiety and negative emotions^9^. Meanwhile, the amygdala may also be involved in pain, traumatic brain injury and cognitive decline^10–14^. In recent years, the relationship between the amygdala and anesthesia has attracted more and more attention. For example, some new studies show that anesthetics activate an endogenous analgesia neural ensemble in the central nucleus of the amygdala^15,16^. Another study suggests that abnormal electrical activity in the amygdala may be a possible mechanism by which anesthesia leads to decreased associative learning and memory^17^. Therefore, we speculate that the amygdala may also play an important role in the process of memory decline caused by anesthesia. However, until now, no similar study has been carried out in the Chinese population.

Alzheimer’s disease (AD) is one of the most common neurodegenerative diseases and the most common form of dementia, with extremely complex pathologies^18^. Despite a number of risk factors have been proposed, causality has not been established^19^. Exposure to anesthetics has been demonstrated to promote pathogenesis of AD in both in vitro and in vivo studies^20,21^. However, some epidemiological surveys have shown conflicting results, for example, a systematic review and meta-analysis showed demonstrated a significant positive association between surgery with general anesthesia and AD^22^, while another systematic review and meta-analysis showed that a history of exposure to surgery with general anesthesia will not associated with an increased risk of AD^23^. Moreover, to date, no clinical trials have addressed the link between exposure to surgery with general anesthesia and the development of AD in humans^24^.

Therefore, whether exposure to surgery with general anesthesia (GA) increases the development of AD remains an open question, and need long-term prospective, randomized clinical studies to examine the relationship between surgery with general anesthesia and AD. In order to solve the above problems, we will use two large longitudinal cohort studies specifically to investigate the association between surgery with general anesthesia and future cognitive impairment, and intend to use structural magnetic resonance of T1 phase to explore the possible mechanism of cognitive decline induced by surgery with general anesthesia. We hypothesize two things: (1) general anesthesia increases the risk for future cognitive decline; and (2) structural changes in the structure of the amygdala may play an important regulatory role in the process of anesthesia-induced cognitive decline.

## Materials and methods^25^

### Participants

Cohort 1 was part of the China Longitudinal Aging Study (CLAS), which has been described in detail previously^26^. There were 1545 elderly people with normal cognitive function involved in the present study, who underwent a screening process that included physical examination, medical history, baseline and 1-year follow-up assessments of cognitive function by a face-to-face interview. According to the follow-up results, of the 1545 elderly people with normal cognitive function, 127 transformed into mild cognitive impairment (MCI), 27 into dementia, and 1391 still maintained normal cognitive function. Then we assigned all cases of cognitive decline (including MCI and dementia) into the cognitive decline group, while those whose cognitive function remained normal were classified as normal group.

Cohort 2 were 194 elderly people with normal cognitive function from a cohort of the Shanghai Brain Aging study. Compared with the first cohort, all participants in the second cohort completed baseline T1-phase MR imaging scans and a series of neuropsychological tests including Montreal Cognitive Assessment (MoCA) (assessing overall cognitive function)^27^, Digit span (assessing attention and short-term memory)^28^, Auditory word learning test (assessing auditory memory)^29^, Associative learning test (assessing association ability and reaction speed), Visual recognition function test (assessing the ability to visually distinguish numbers, letters, and words), Verbal fluency tasks (assessing Language ability, semantic memory and executive function)^30^, Webster’s mapping, and Wechsler block diagram (assessing executive function)^31^. All scales were performed by professionally trained psychological testers, with consistency training to ensure the accuracy and consistency of the scale assessment. However, they did not undergo a follow-up evaluation.

All the subjects had signed informed consent before this study was initiated and ethical approval was obtained from the ethics committee of Shanghai mental health center.

### Exposure

Self-reported surgical and anesthesia data were collected through interviews at baseline. Participants were asked, "Have you ever undergone surgery under general anesthesia”, If so, participants reported detailed information, including the age of surgery, and type of surgery. The main drugs used for general anesthesia include enflurane, isoflurane, morphine and so on.

### Outcomes

Dementia and mild cognitive impairment (MCI) were diagnosed according to the Diagnostic and Statistical Manual of Mental Disorders, 4th Edition (DSM-IV) and revised Petersen’s diagnostic algorithm^32^, respectively. We defined the onset date of dementia and MCI as the intermediate date between the visit that triggered the assessment resulting in a positive diagnosis and the previous study visit. At the same time, all of the impaired participants completed the daily Living Ability Scale. Patients with MCI had to have no decline in daily living ability, but patients with dementia had to have a significant decline in daily living ability^33^.

### Covariates

General demographic information (including age, gender, education), daily living information (including smoking history, consumption of alcohol), as well as disease information (such as hypertension and diabetes) were collected by a standardized questionnaire. Those variables that differed between surgery and non-surgery groups were considered as covariates.

### T1 phase structure magnetic resonance imaging

The brain structure images were captured using the Magnetom Verio 3.0 T scanner (Siemens, Munich, Germany). The rapid gradient echo (MPRAGE) sequence parameters prepared by T1-weighted THREE-DIMENSIONAL magnetization were as follows: TR = 2,300 ms, TE = 2.98 ms, matrix size = 240 × 256; flip angle of 9 degrees, slice thickness = 1.2 mm, field of view (FOV) = 240 × 256 mm. Volume data was evaluated by automated procedures, as described by Wolz et al.^34^. Based on our previous research^35,36^ and the research hypothesis in the introduction, volume and asymmetry of amygdala, and brain size index were extracted for each subject (using FreeSurfer). In addition, to evaluate the effect of left–right difference, the asymmetry index was calculated by the formula [right volume-left volume]/[Total volume] × 100%. Quality control is done by overlapping output parcellations on FreeSurfer’s templates and performing visual evaluations to ensure registration and parcell quality.

### Ethical approval and consent to participate

The Research Ethical Committee of the affiliated mental health center of Shanghai jiaotong university school of medicine approved this study, and written informed consent was obtained from all participants before the study. The study was performed in accordance with The Code of Ethics of the World Medical Association (Helsinki Declaration of 1964, as revised in 2018).

## Statistical analysis

Continuous variables were expressed as mean ± standard deviation (SD), and categorical variables were expressed as frequencies (%) (Cohort 1 and 2). To test whether data conforms to normal distribution, we applied a single sample Kolmogorov–Smirnov test (Cohort 1 and 2). Next, we used independent sample t-test and Kruskal–wallis H to compare the normal data (MoCA and education) and non-normal data (age, other neurological tests and amygdala volume) between the surgery group and the non- surgery group, respectively (Cohort 1 and 2). And we also used Chi-square tests to compare those classification variables (Cohort 1 and 2). Then Cox regression analysis was used to further explore the relationship between surgery with general anesthesia and cognitive change (controlled for other relevant variables) (Cohort 1) and partial correlation analysis was used to explore the correlation between surgery with general anesthesia and brain structure (Cohort 2). Linear regression analysis (mediating model) and correlation analysis were also used to investigate the association between surgery with general anesthesia, cognitive related brain areas, and cognitive scores (Cohort 2). Two-tailed tests were used at a significance level of *P* < 0.05 for all analyses. The data was analyzed using SPSS 22.0 (IBM Corporation, Armonk, NY, USA).

## Results

### Characteristic of subjects with different surgical states (Cohort 1)

Overall, those with a history of surgery were older, and a larger proportion were women, but a smaller proportion were smokers (*p* < 0.05), while there was no statistical difference (*p* > 0.05) in education, drinker, hypertension and diabetes between the two groups. Table 1 shows the results.

**Table 1:** Baseline characteristics by surgery in 1545 older people.

Characteristics	Surgery (n = 667)	Non-surgery (n = 878)	X^2^ OR T	*P*
Age, y	71.08 ± 7.34	69.79 ± 7.67	3.094	0.002*
Education, y	9.66 ± 5.157	9.27 ± 4.993	1.445	0.149
Male, n (%)	290(43.5)	462(52.6)	12.679	< 0.001*
Smoker, n (%)	166(24.9)	277(31.5)	8.224	0.004*
Drinker, n (%)	125(18.7)	184(21.0)	1.163	0.304
Hypertension, n (%)	334(50.1)	395(45.0)	3.935	0.051
Diabetes, n (%)	112(16.8)	116(13.2)	3.861	0.051
Anesthesia related information				
Course of disease, y 23.46 ± 17.34				
Isoflurane, n (%) 20(3.0)				
fentanyl, n (%) 233(34.9)				
Others, n (%) 414(62.1)				

*Means *p* < 0.05.

### The results of the Multiple Cox regression model (Cohort 1)

**Figure 1:**
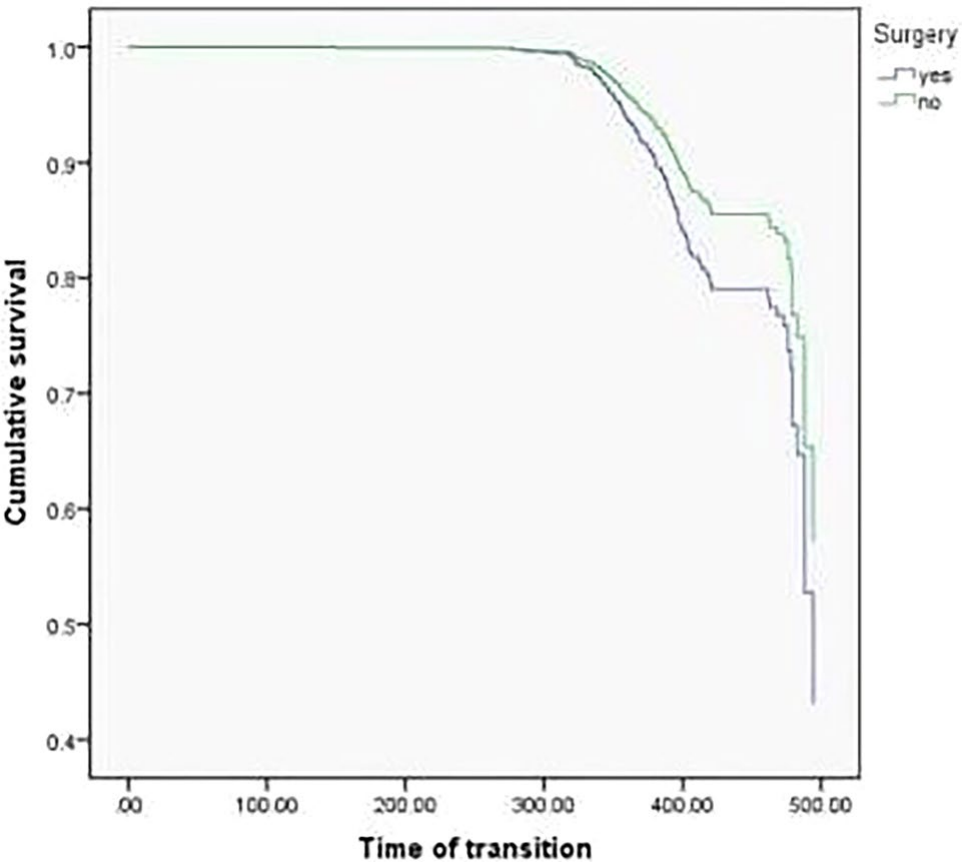
Comparison of the risk of future cognitive impairment between patients undergoing general anesthesia and non-surgical controls.

Multiple Cox regression model was used to explore the relationship between surgery with general anesthesia and future cognitive decline (Cognitive decline was regarded as the dependent variable, and transition time was taken as the time variable). Model 1 did not control any variables, and the results showed that surgery with general anesthesia was a risk factor for cognitive decline (*p* = 0.013, HR = 1.506, 95% CI 1.091–2.078); Model 2 controlled some variables, such as age and gender, and Model 3 furtherly controlled other variables, such as smoking, and different statistical models still did not change the statistical results (Table 2). The results of the survival curve suggested that older adults with a history of surgery with general anesthesia would develop cognitive impairment earlier and more often (*p* = 0.013, HR = 1.506, 95% CI 1.091–2.078). Figure 1 presents the results. However, we did not find any effect of the duration of anesthesia or the choice of anesthetic agent on cognitive outcomes.

**Table 2:** Results of COX regression analysis.

Variables	B	S.E	Wald	df	*p*	HR	95% confidence interval	
Model 1								
Surgery	0.409	0.164	6.189	1	0.013*	1.506	1.091	2.078
Model 2								
Surgery	0.430	0.165	6.770	1	0.009*	1.538	1.112	2.127
Age	− 0.425	0.072	35.004	1	< 0.001*	0.654	0.568	0.753
Male	− 0.221	0.175	1.594	1	0.207	0.802	0.570	1.130
Model 3								
Surgery	0.427	0.165	6.652	1	0.010*	1.532	1.108	2.119
Age	− 0.435	0.072	36.566	1	< 0.001*	0.647	0.562	0.745
Male	− 0.025	0.212	0.014	1	0.905	0.975	0.643	1.478
Smoking	0.361	0.238	2.289	1	0.130	1.434	0.899	2.288

Model 1 contains only surgery.

Model 2 contains surgery, age and gender.

Model 3 contains surgery, age, gender and smoker.

*Mean *p* < 0.05.

### The connection between surgery with general anesthesia and brain structure (Cohort 2)

**Figure 2:**
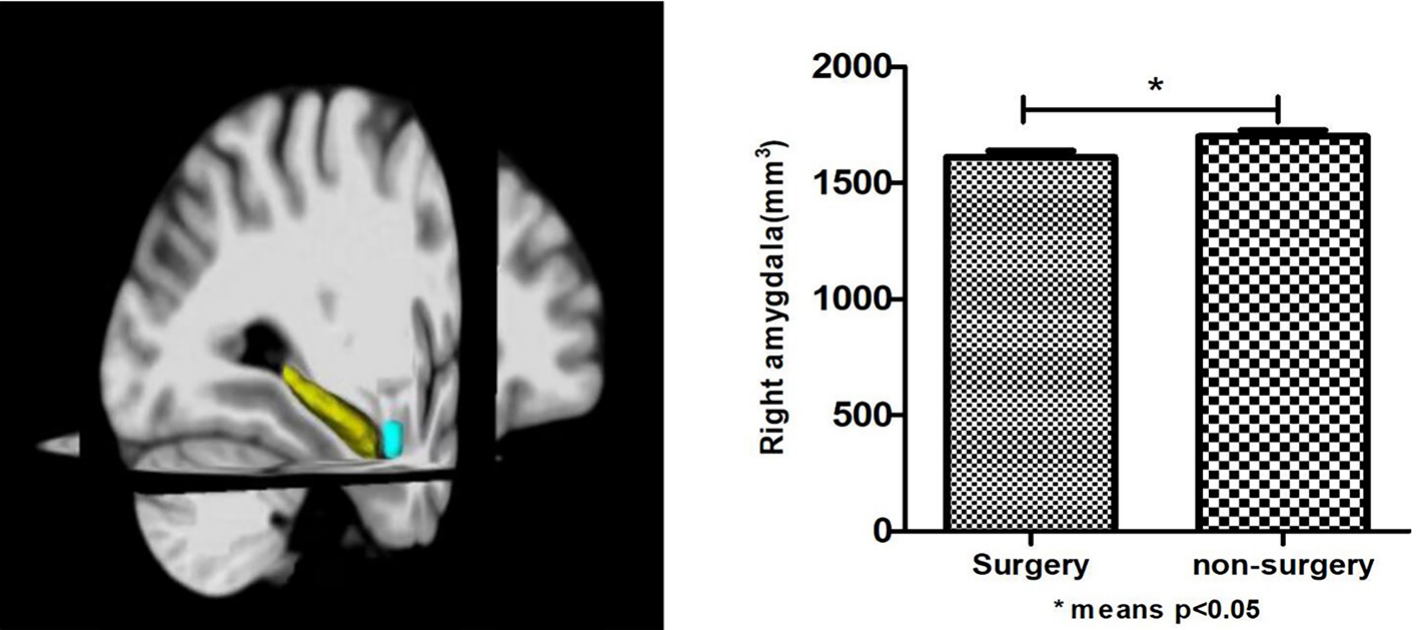
Comparison of the volume of the right amygdala in patients undergoing general anesthesia and non-surgical controls.

**Figure 3:**
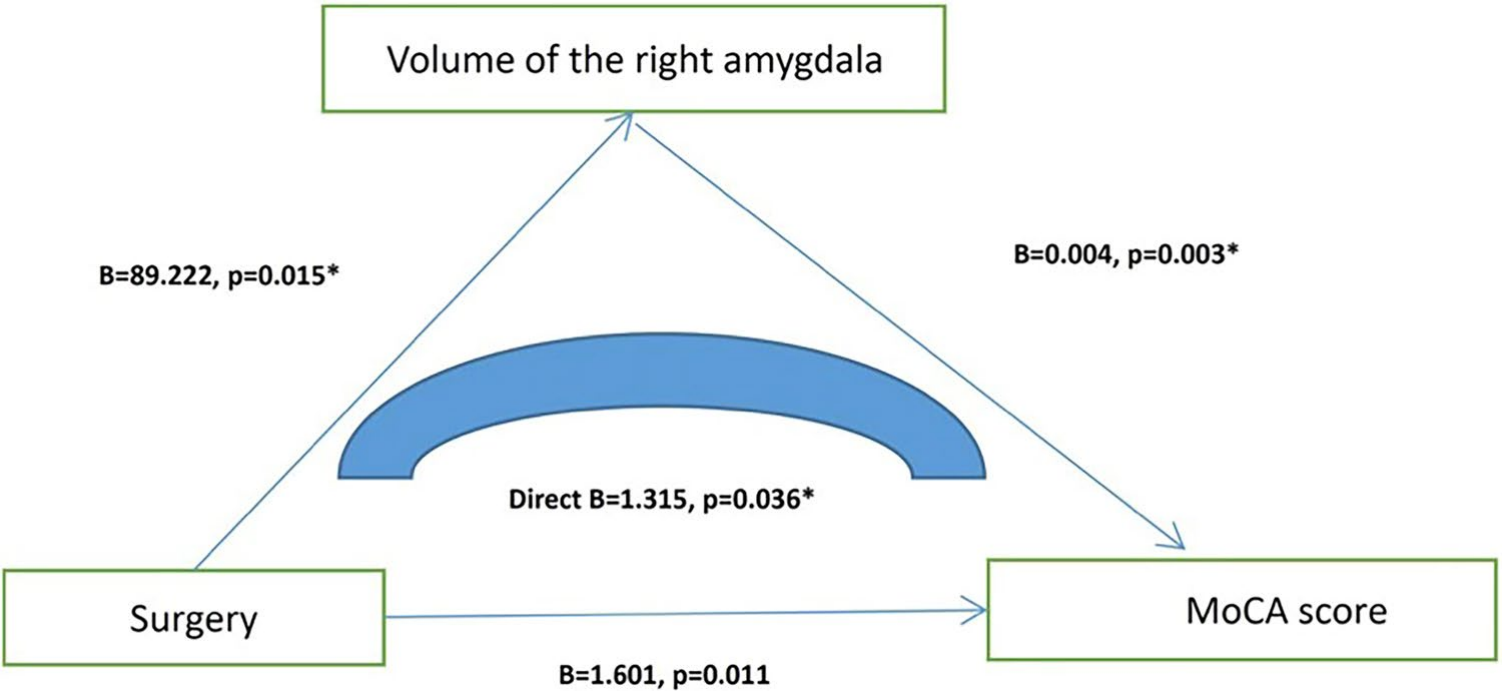
There was an intrinsic relationship between general anesthesia, right amygdala volume, and MOCA score.

To explain the possible mechanisms by which surgery with general anesthesia affects cognition, we added structural magnetic resonance data. Based on whether they had a history of surgery with general anesthesia, these 194 people were divided into surgery group (n = 92) and non-surgery (n = 102) group (Table 3). We ultimately found that older adults with a history of surgery with general anesthesia had lower MoCA scores and smaller right amygdala volume (*p* < 0.05) (Fig. 2). Through correlation analysis, we found that the volume of the right amygdala was significantly correlated (*p* = 0.003, r = 0.212) with MoCA. Then by using the linear regression analysis (mediation model), we found that surgery with general anesthesia directly affected the MoCA score by affecting the volume of the right amygdala (B = 1.315, *p* = 0.036 95% CI 0.088–2.542) (Fig. 3).

**Table 3:** Comparison of general demographic data between the surgery group and the non-surgery group.

Variables	Surgery (n = 92)	Non-surgery (n = 102)	X^2^ or t	*p*
Age, y	70.02 ± 7.206	68.52 ± 7.470	1.422	0.157
Education, y	8.44 ± 4.452	9.51 ± 3.601	− 1.814	0.071
Male, n (%)	39(42.4)	57(55.9)	3.522	0.064
Smoker, n (%)	26(28.3)	32(31.4)	0.223	0.754
Drinker, n (%)	16(17.4)	22(21.6)	0.536	0.476
Tea drinker, n (%)	36(39.1)	42(41.2)	0.084	0.883
Take exercise, n (%)	56(60.9)	68(66.7)	0.705	0.455
Hobby, n (%)	60(65.2)	64(62.7)	0.128	0.766
Hypertension, n (%)	44(47.8)	51(50.0)	0.091	0.776
Diabetes, n (%)	17(18.5)	19(18.6)	0.001	1.000
Neuropsychological tests				
MoCA	23.34 ± 4.983	24.94 ± 3.612	− 2.528	0.012*
Digit span	14.60 ± 4.299	15.11 ± 3.857	− 0.888	0.376
Auditory word learning test	32.23 ± 8.982	32.17 ± 9.320	0.045	0.964
Associative learning test	6.55 ± 3.407	6.58 ± 3.169	− 0.072	0.943
Visual recognition function test	3.41 ± 0.869	3.62 ± 0.630	− 1.912	0.058
Language fluency	26.98 ± 8.226	28.58 ± 9.771	− 1.225	0.222
Webster’s mapping	10.47 ± 3.53	10.83 ± 4.483	− 0.627	0.532
Wechsler block diagram	27.26 ± 8.097	27.89 ± 8.034	− 0.554	0.587
Brain structure				
Total brain volume, cm^3^	1443.68 ± 149.390	1464.90 ± 145.76	− 0.996	0.321
Left amygdala, cm^3^	1.479 ± 0.237	1.527 ± 0.235	− 1.414	0.159
Right amygdala, cm^3^	1.611 ± 0.257	1.700 ± 0.251	− 2.445	0.015*

*Means *p* < 0.05; *MMSE* mini-mental state examination, *MoCA* Montreal cognitive assessment.

## Discussion

The aim of this study was to explore the association between surgery with general anesthesia and cognitive decline and to explore the possible mechanisms by which surgery with general anesthesia affects cognitive function. By using two large cohort studies, we found that (1) surgery with general anesthesia was a risk factor for cognitive decline; (2) surgery with general anesthesia may affect cognitive function by affecting the volume of the right amygdala, and there might be a mediating effect between the three factors (surgery with general anesthesia, right amygdala and cognitive function). Through this study, we may be able to answer the very complex question of why general anesthesia causes cognitive decline, and provide a more reliable basis for subsequent prevention and intervention.

There is a strong belief that elderly people who undergo major surgery, particularly heart surgery, are at risk of cognitive decline lasting more than six months^37,38^. However, since anesthesia and surgery are often used together, it is not possible to tell which factor plays a greater role in the process of cognitive decline. Based on laboratory and previous evidence, we hypothesize that both surgery and general anesthesia can precipitate incident dementia^39^. In our current study, we used two community cohorts to investigate the association and possible mechanisms between surgery with general anesthesia and future cognitive decline, and we ultimately found that surgery with general anesthesia was a risk factor for cognitive decline, independent of gender, age, and education. There is growing awareness that surgical or anesthetic drugs exposure might be associated with temporary or permanent cognitive impairment, but the results are inconsistent^40,41^. For example, Mincer et al. found that elderly people may take longer than younger adults to achieve cognitive recovery after immediate anesthesia, but all will fully recover to baseline within 30 days^42^, while in Juraj Sprung et al. study, they found that exposure to general anesthesia for procedures at age ≥ 40 years was not associated with prevalent mild cognitive impairment (MCI) in the elderly^43^. Therefore, future clinical studies should strictly limit the diagnostic tools, evaluation programs and inclusion criteria for cognitive impairment, so as to achieve relatively consistent research conclusions.

To further explore the possible mechanisms by which surgery with general anesthesia affects cognitive functions, we then included magnetic resonance data. Based on our previous research, we still mainly focused on the effects of amygdala on cognitive function^35^. Using data from 194 elderly people, matched for age, sex, and education, we found that elderly people with a history of general anesthesia had smaller right amygdala volume and worse MoCA scores. Through correlation analysis, we found that the volume of the right amygdala was positively correlated with the total MoCA score. Then, by using the linear regression analysis (mediation model), we found that surgery with general anesthesia directly affected the MoCA score by affecting the volume of the right amygdala. In previous cognitive studies, MoCA is usually used as a screening tool for overall cognitive function, and the score usually reflects the overall cognitive level of an individual. Based on our results and statistical analysis, it is possible to conclude that there may be an association between general anesthesia, amygdala, and overall cognitive function, however, the above conclusions still need to be further verified by longitudinal studies.

It has been proposed that the human amygdala may not only encode the emotional value of sensory events, but more generally mediate assessments of their relevance to personal goals, including relevance to action or task-based needs^44^. Moreover, the amygdala has also been shown to regulate additional cognitive processes, such as attention or memory^45^. Some scholars have proposed that early atrophy and dysfunction of the amygdala and hippocampus may develop into biomarkers for Alzheimer’s disease^35,46^. Lan et al.^47^ found that elderly patients with knee osteoarthritis (KOA) experienced a decrease in functional connectivity (decreased low frequency fluctuation amplitude (ALFF) in left precuneus gyrus and middle temporal gyrus) in certain brain regions after total knee arthroplasty (TKA). In the study by Limor Avivi-Arber, they found that tooth extraction in adult female mice was associated with significant reductions in cortical regions and voxel volume, which are involved in motor, cognitive, emotional, and processing somatic sensors, as well as increased volume in the subcortical sensorimotor and temporal-limbic frontal regions (including the amygdala). Therefore, combined with previous studies and our current study, we hypothesized that the amygdala may be a sensitive brain region during general anesthesia and a key location for cognitive decline.

## Limitations

There are some limitations in our study. Firstly, our study was only a cross-sectional study and was unable to establish a causal link between general anesthesia and cognitive decline; Secondly, a relatively small sample size will decrease study reliability. Thirdly, there are many confounders in our study, such as different types of surgery, different choices of anesthetic drugs and different anesthetic durations, which may affect the results of the study to some degree. Fourth, our current study focused solely on the relationship between the amygdala and general anesthesia, but did not account for the impact of other cognitive brain regions (such as the hippocampus) on the findings, which is perhaps the greatest limitation of our study. Therefore, in the future research, we will focus on addressing these issues.

## Conclusions

Surgery with general anesthesia may increase the risk of cognitive decline, and its mechanism may be related to its effect on the volume of the right amygdala.

## Data Availability

The datasets used and/or analysed during the current study available from the corresponding author on reasonable request.
